# Longitudinal analysis on parasite diversity in honeybee colonies: new taxa, high frequency of mixed infections and seasonal patterns of variation

**DOI:** 10.1038/s41598-020-67183-3

**Published:** 2020-06-26

**Authors:** Carolina Bartolomé, María Buendía-Abad, María Benito, Beatriz Sobrino, Jorge Amigo, Angel Carracedo, Raquel Martín-Hernández, Mariano Higes, Xulio Maside

**Affiliations:** 1 Grupo de Medicina Xenómica, CIMUS, Universidade de Santiago de Compostela, 15782 Santiago de Compostela, Galicia Spain; 2 Instituto de Investigación Sanitaria de Santiago (IDIS), 15706 Santiago de Compostela, Galicia Spain; 3 Instituto Regional de Investigación y Desarrollo Agroalimentario y Forestal (IRIAF), Laboratorio de Patología Apícola, Centro de Investigación Apícola y Agroambiental (CIAPA), Consejería de Agricultura de la Junta de Comunidades de Castilla-La Mancha, 19180 Marchamalo, Spain; 4 Fundación Pública Galega de Medicina Xenómica, Servicio Galego de Saúde (SERGAS), 15706 Santiago de Compostela, Spain; 5 Instituto de Recursos Humanos para la Ciencia y la Tecnología, Fundación Parque Científico Tecnológico de Albacete, 02006 Albacete, Spain; 6 Departamento de CC. Forenses, Anatomía Patolóxica, Xinecoloxía e Obstetricia, e Pediatría, Universidade de Santiago de Compostela, 15782 Santiago de Compostela, Galicia Spain

**Keywords:** Parasitology, Ecological epidemiology

## Abstract

To evaluate the influence that parasites have on the losses of *Apis mellifera* it is essential to monitor their presence in the colonies over time. Here we analysed the occurrence of nosematids, trypanosomatids and neogregarines in five homogeneous colonies for up to 21 months until they collapsed. The study, which combined the use of several molecular markers with the application of a massive parallel sequencing technology, provided valuable insights into the epidemiology of these parasites: (I) it enabled the detection of parasite species rarely reported in honeybees (*Nosema thomsoni, Crithidia bombi, Crithidia acanthocephali*) and the identification of two novel taxa; (II) it revealed the existence of a high rate of co-infections (80% of the samples harboured more than one parasite species); (III) it uncovered an identical pattern of seasonal variation for nosematids and trypanosomatids, that was different from that of neogregarines; (IV) it showed that there were no significant differences in the fraction of positive samples, nor in the levels of species diversity, between interior and exterior bees; and (V) it unveiled that the variation in the number of parasite species was not directly linked with the failure of the colonies.

## Introduction

The activity of the European or Western honeybee, *Apis mellifera*, is crucial for ensuring food production and maintaining the biodiversity of the ecosystems worldwide^1^. During the last decades, regional losses of honeybee colonies have been frequently reported^2–5^, although at a global scale the number of managed colonies seem to have risen, either due to their high demand for honey production and pollination services in certain areas^6,7^ or to the lack of adequate tools to quantify their mortality on a harmonized basis^8^.

The long-term declines observed in local stocks of managed honeybees is usually attributed to politic and socio-economic factors^7,9^, whereas annual losses (which are often amended by beekeepers replacing lost colonies) respond to a wider spectrum of causes^4,5,8,9^. These are multiple and interlinked, and go from degradation of the ecosystems due to global warming (e.g. weather events) and changes in land use (e.g. urbanisation, intensive agriculture), to health issues (e.g. stress due to beekeeping management, pollution entailed by the use of agrochemicals or dispersal of native and exotic parasites)^1,2,8–20^.

Given that the environment provides an optimal scenario for parasite transmission among social insects^15,21–24^, and taking into account that the fate of the colonies may depend on the timing and sequence of infections^25^, it is important to survey if the occurrence and diversity of these agents vary over time^26–31^.

Nowadays, the most comprehensive and cost-effective mode of assessing parasite diversity is to apply massive parallel sequencing, which allows the simultaneous identification of multiple pathogens across large numbers of samples, overcoming the need for cloning prior to Sanger sequencing, which is expensive and time-consuming^32^. Following this approach, we analysed the diversity of three major groups of unicellular parasites (nosematids, trypanosomatids and neogregarines) in five homogeneous colonies that were screened sequentially until their death.

## Results and Discussion

Here we describe the results of a longitudinal study of the presence of parasites of the groups Nosematidae, Trypanosomatidae and Neogregarinorida in five homogenous colonies, which were monitored since the last trimester of 2014 until their collapse (the latest in August 2016).

### Molecular detection of parasites

PCRs with specific primers allowed the detection and molecular identification of parasites in 76 of the 80 pooled DNA samples (95%; Table 1 and Supplementary Table S1). Nosematids were the most prevalent group (76.3%), followed by trypanosomatids and neogregarines (72.5% and 33.8%, respectively).

**Table 1:** Relative frequencies of parasites in the samples, expressed as percentage.

Parasite group	Species	Total (*N* = 80)
Nosematids	*Nosema ceranae*	76.3
	*Nosema thomsoni*	2.5
	All	76.3
Trypanosomatids	*Crithidia acanthocephali*	22.5
	*Crithidia bombi*	53.8
	*Crithidia mellificae*	61.3
	*Lotmaria passim*	68.8
	Trypanosomatidae sp.	1.3
	All	72.5
Neogregarines	*Apicystis bombi*	32.5
	Neogregarinorida sp.	27.5
	All	33.8

The primers targeting the *Actin* and *RPB1* (*RNA polymerase II large subunit RPB1*) loci of nosematids and trypanosomatids, respectively, displayed higher sensitivity (96.7% and 84.5% of the positive samples) than the ones designed for the *SSU* (*small-subunit ribosomal DNA*) loci (78.7% and 55.2%, respectively; Supplementary Table S1). The rate of coincidence between the two markers was 75.4% for nosematids (i.e. both primers sets produced a band of the expected size in 46 out of 61 of the positive samples) and substantially lower for trypanosomatids (39.7%). This means that the PCR approach systematically misses a fraction of the positive samples, so that the results might underestimate the true parasite prevalence. To minimize this effect, in this study the presence of a parasite was determined by a positive result with either marker in a given sample.

The choice of primers had an effect on the sensitivity but also on the variety of species detected. Indeed, the pair targeting the *Actin* locus allowed the detection of *Nosema ceranae* and *Nosema thomsoni*, whereas the one targeting the *SSU* only permitted the identification of *N. ceranae* (Supplementary Table S1). Similarly, the primers used to amplify *RPB1* revealed the presence of *Lotmaria passim*, *Crithidia mellificae, Crithidia bombi, Crithidia acanthocephali* and a new undescribed taxon (Trypanosomatidae sp.), while those amplifying the *SSU* only allowed the identification of *L. passim* and *C. bombi*. This lower performance of *SSU* is rather surprising as the amplification of ribosomal genes is one of the gold standards for molecular barcoding due to their sequence conservation and large number of copies. Nevertheless, the amplification success of *a priori* universal primers might be influenced by multiple factors like the GC-content within the amplified region, the number of mismatches between species^33^, or even the targeted species^34^, as the presence of previously undetected polymorphisms within the priming sites could drastically reduce their efficiency. These results emphasize the convenience of using a multilocus approach in order to produce a more accurate description of the diversity patterns of honeybee parasites, as many of them would have gone otherwise unnoticed.

### Parasite diversity

*N. ceranae* was the most frequent pathogen of the series and was found in all *Nosema* positive samples. This is in line with previous epidemiologic analyses carried out in the same area^31,35^, whereas *N. thomsoni* was detected along with *N. ceranae* in two isolates (PR-05 I and PR-21 F; Supplementary Table S1). *N. thomsoni* was first described infecting a moth, *Choristoneura conflictana*^36^, and has now been identified in many other insect species, including bumblebees, beet webworms, ladybirds and solitary bees^37–41^. To the best of our knowledge, this is the first time it is reported in honeybees, although its presence in these pollinators might not be necessarily associated with tissue infection, as shown by Pereira *et al*.^42^ in bumblebees.

It must be highlighted that none of the samples tested positive for other nosematids previously detected in *A. mellifera*, like *Nosema apis*^43^ or *Nosema neumanni*^44^. The only known sequence of the latter taxon corresponds to the *SSU* locus, and it contains a mismatch in the region complementary to the forward primer used here that might have hindered its amplification.

**Figure 1:**
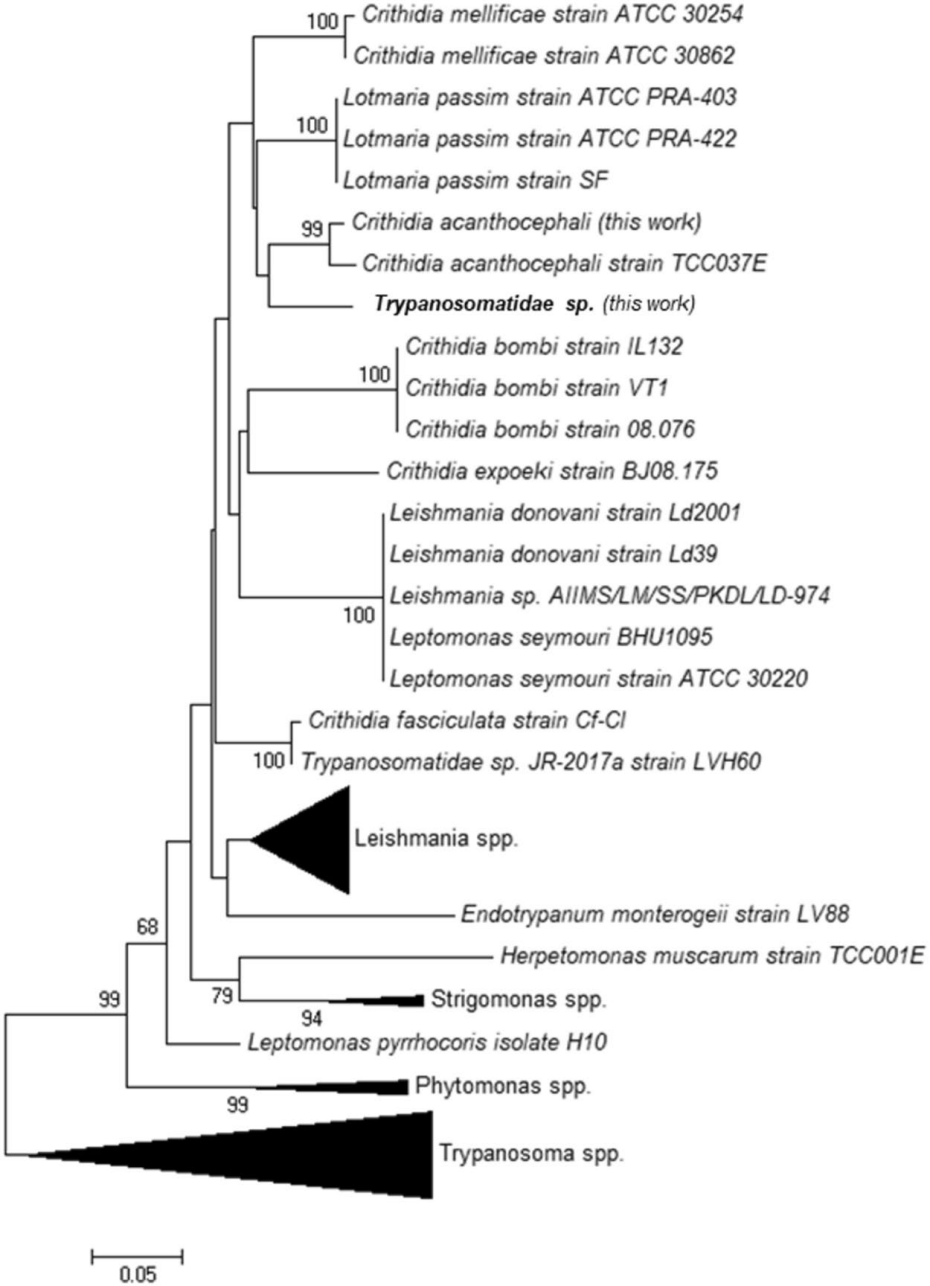
Neighbor-joining phylogeny (*RPB1* sequences) showing the evolutionary relationships of trypanosomatid species, including the new taxon detected in this work (Trypanosomatidae *sp*.; highlighted in bold).

Trypanosomatids displayed the highest species diversity, with an average of 2.1 ± 0.32 species per sample (Supplementary Table S1). In agreement with prior studies^35,45–47^, *L. passim* was the most prevalent trypanosomatid species of the dataset (68.8%; Table 1), closely followed by *C. mellificae* (61.3%) and *C. bombi* (53.8%), which are usually found at much lower rates of prevalence^45,48^. In fact, until now *C. bombi* was very seldom reported in honeybees^21,49^. It is also interesting to note the first identification of *C. acanthocephali*^50^ in *Apis mellifera*, with an occurrence of 22.5%, as well as the detection of a putatively new taxon, *Trypanosomatidae sp*. (GenBank accession number MN038411) which, according to the phylogeny constructed with other trypanosomatid *RPB1* sequences, is closely related to *C. mellificae*, *C. acanthocephali* and *L. passim* (Fig. 1). However, the synonymous divergence between this taxon and the later species estimated at the *RPB1* locus was 49.6%, 42.9% and 50.4%, respectively, which suggests that it likely corresponds to a different species.

The finding of these and other species rarely detected in honeybees, like C*. bombi* or *C. acanthocephali*, suggests that trypanosomatids may have a broader spectrum of hosts than previously reported, although their status as incidental vectors or natural reservoirs, where they may cause a disease, is still to be clarified. Besides, given that whole abdomens were used for DNA extraction, we cannot rule out that the presence of these and other novel species could represent an external contamination of the exoskeleton by flower-visiting rather than a true gut infection.

**Figure 2:**
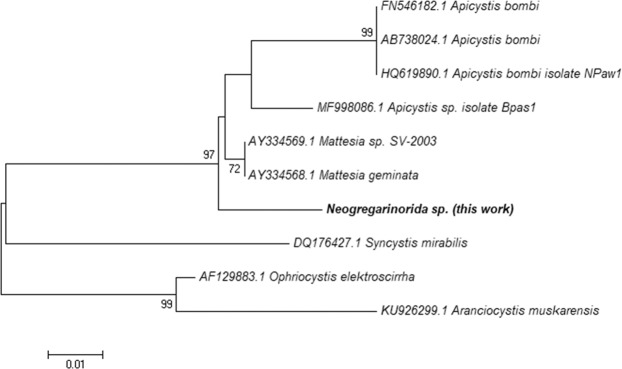
Neighbor-joining phylogeny (*SSU* sequences) showing the evolutionary relationships of neogregarine species, including the new taxon detected in this work (Neogregarinorida sp; highlighted in bold).

The massive parallel sequencing approach used here also allowed the detection of two Neogregarinorida species, *Apycistis bombi* and a second taxon (GenBank accession number MN031271) that was nearly as frequent as the former (Table 1). Its phylogenetic position within the group is displayed in Fig. 2 and the estimates of silent nucleotide divergence between this new taxon and *A. bombi* and *Mattesia* spp. were 4.6% and 2.3%, respectively, which are of the same magnitude as those observed between other neogregarine species. However, it must be emphasized that the *SSU* is not ideal to infer taxonomic relationships among gregarine species, which show very heterogeneous rates of evolution at this locus^51^.

The analyses of diversity showed that parasites of the same groups were found in the same sample more often than would be expected assuming random expectations (Table 2). In principle such an effect could be attributed to the lack of stochasticity of parasites present in consecutive samples. For instance, if a parasite persists for long periods of time in a colony (low turnover) its presence at any given point in time would make its detection more likely in the following samples. In addition, parasites would tend to be associated with other species found in the preceding samples. But in contrast with these predictions, the observed surplus of co-infections was only detected amongst species of the same group (specifically amongst trypanosomatids and neogregarines) but not between species of different groups. In fact, no significant pairwise associations were observed between *N. ceranae, L. passim*, and *A. bombi*, the three species with lower turnover rates of their groups (approximated as the inverse of the mean number of consecutive positive samples; Supplementary Table S2). These observations argue against the hypothesis of a correlation in parasite composition between consecutive samples and suggest a role for other parasite group-specific factors in modelling the patterns of infection over time (Supplementary Table S3).

**Table 2:** Chi-square of goodness of fit tests of the observed frequency of coinfections between pairs of parasite species as compared with random expectations.

	*L. passim*		*C. bombi*		*C. mellificae*		*C. acanthocephali*		*N. ceranae*		*A. bombi*	
*C. bombi*	36.7	***										
*C. mellificae*	26.7	***	22.8	***								
*C. acanthocephali*	7.1	**	4.4	*	8.1	**						
*N. ceranae*	1.2	ns	0.0	ns	0.0	ns	0.3	ns				
*A. bombi*	0.0	ns	0.2	ns	1.2	ns	1.3	ns	0.7	ns		
Neogregarinorida sp.	0.3	ns	0.8	ns	0.3	ns	0.9	ns	1.3	ns	31.5	***

Note: the presence of a parasite in a colony at a given sampling date was determined by its detection either in interior, exterior bees, or both; *P < 0.05; ** *P* < 0.01; *** *P* < 0.001; ns = non-significant.

### Seasonal analysis of parasite diversity

Eighty percent of the samples harboured more than one parasite and the average number of parasite species detected per sample was 3.5 ± 0.20 (Supplementary Table S4). No significant differences between interior and exterior bees were observed. These results go in line with previous recent data^49^ and suggest that mixed infections are the norm rather than the exception in honeybees^28^.

**Figure 3:**
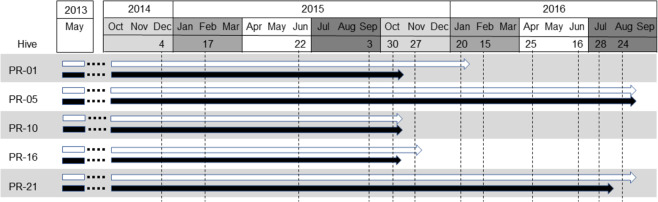
Representation of the collection scheme used for Ion PGM sequencing. Sampling dates are indicated (dotted lines). Interior and exterior honeybees are represented by white and black arrows, respectively.

Considering that environmental factors are thought to have a strong influence on the parasite composition of the colonies^26^, we analysed the seasonal variation of parasite frequency and diversity in the five colonies over time. Samples were grouped by season according to the sampling scheme detailed in the Experimental Procedures section (Fig. 3).

**Figure 4:**
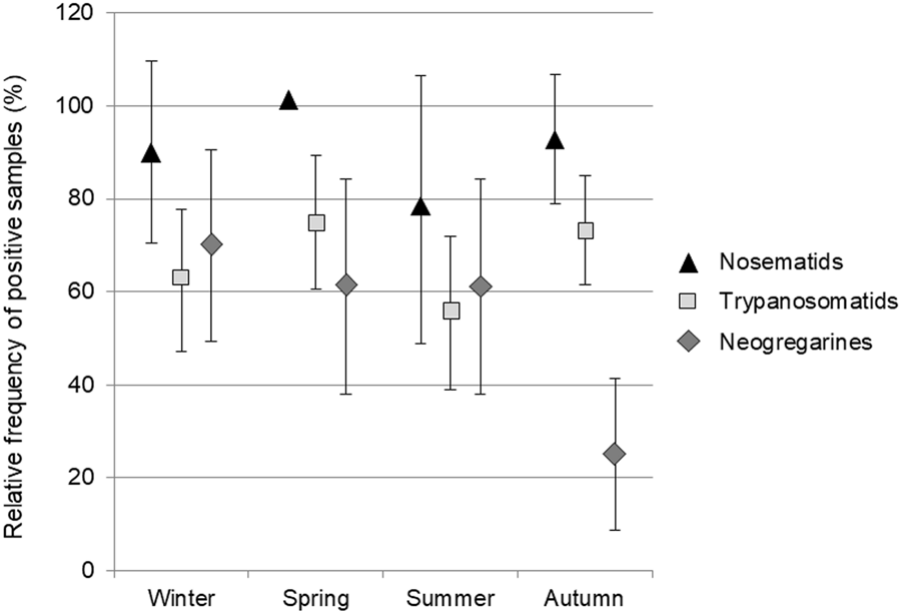
Mean fraction of positive samples (%) across seasons. Raw data as in Supplementary Table S5; *N. thomsoni* and Trypanosomatidae sp. were excluded due to their low frequency. Error bars represent 95% confidence intervals.

Nosematids and trypanosomatids displayed a nearly identical pattern of seasonal variation with slight fluctuations that reached the highest frequencies during spring and the lowest in summer (Fig. 4). The strong within-group associations described above meant that most species of these groups shared this seasonal pattern (Supplementary Table S5), with punctual deviations such as *C. bombi*, which was slightly less prevalent in winter, or *C. acanthocephali*, which was more common in autumn. Current samples sizes limited our ability to determine the statistical significance of these within-group variation.

These trends were consistent along the two consecutive complete annual cycles (autumn-winter-spring-summer) included in the study (Fig. 3) and suggest that, although nosematid and trypanosomatid species could be found all year round, they find it harder to overcome the hot dry summers in the Iberian Meseta, with average daily high temperatures over 30 °C and monthly rainfall below 20 mm in July and August. These climatic conditions, together with the simultaneous increase in the number of individuals in the colonies^52^, contribute to explain the reduction in occurrence of these parasites in summer, which was already observed in previous reports based either on prevalence data (e.g. Stevanovic *et al*., 2013^53^ and Gisder *et al*., 2017^54^ for *N. ceranae*; or Glenny *et al*., 2017^26^ for *L. passim*) or parasite loads (e.g. Higes *et al*., 2008^52^ and Chen *et al*., 2012^55^ for *N. ceranae*; Glenny *et al*., 2017^26^ and D’Alvise *et al*., 2019^56^ for *L. passim*; or Vejnovic *et al*., 2018^30^ for *L. passim* and *N. ceranae*).

Contrastingly, the occurrence of the two neogregarine species followed a distinct pattern (*P* < 0.001 in a Chi-square test of goodness of fit) with little variation from winter through summer and a sharp drop in autumn (Fig. 4 and Supplementary Table S5). This seasonal trend could be related with the change of activity (reduction of foraging and breeding) and progressive reduction of the population of the colony during autumn. Indeed, it has been demonstrated that the foraging activity of adult honeybees has an important influence on their exposure to pathogens, as fruit, flowers and other food sources are frequently contaminated by other pollinators and function as fomites, contributing to the spread of parasites across visiting insect species^15,21,52,57,58^. However, no significant differences were detected in the fraction of positive samples (Supplementary Tables S1 and S6), nor in the levels of species diversity (Supplementary Table S7) or seasonal trends (Supplementary Figure S1) between interior and exterior bees throughout this study, which suggests a very rapid transmission of enteric parasites between the two groups of bees, most likely through mouth-to-mouth transmission of fluids (trophallaxis). Given that our survey involved a limited number of colonies, it would be of great interest to carry out larger studies (including samples from different locations) to verify if their seasonal trends are similar to those described above.

It should be noticed that the variation of species diversity was associated with seasonal changes but it did not display any obvious relationship with the collapse of the colonies. For instance, three colonies collapsed in mid-autumn 2015 and the other two, with no clinical signs by then, died in late summer 2016 (Fig. 3). In addition, high parasite diversity values were already recorded at early stages of the experiment (e.g. between six and seven species were detected in colonies PR-05, PR-10 and PR-16 in the second sampling date; data from Supplementary Table S1). Also, the average number of species per sample across colonies did not reveal any tendency to increase in the five sampling dates prior to their collapse: 5.8, 4.6, 5.6, 4.6 and 2.6, respectively (data from Supplementary Table S1). These results argue against the hypothesis of a build-up of parasite diversity before the colonies’ collapse, which means that the role of these parasites as drivers of the death of the colonies does not probably reside on the actual number of species but on the presence of one or a combination of them. In this regard, *N. ceranae* was the only species under study present in the samples of all colonies prior to their collapse. The pathogenic effects of this parasite on honeybees, which lead to the shortening of the workers’ lifespan and ultimately to the failure of the colonies^52,59^, are well known in Southern Europe where this highly prevalent pathogen is considered a serious threat.

This work was focused on the analysis of parasite diversity from a qualitative point of view, so pathogens were identified but not quantified. Given that parasite load might be an important factor to explain the effect of the infections on colony health, future research should take this aspect into account, as well as extend the screening to additional infectious agents, such as viruses (e.g. deformed wing virus, black queen cell virus, among others), bacteria-like (e.g. *Melissococcus plutonius, Paenibacillus larvae*) and mites (e.g. *Varroa, Acarapis woodi*)^60,61^ in order to get a more comprehensive picture of the influence that these factors (pathogen diversity, load and interactions) may have on colony losses.

## Methods

### Samples and DNA extraction

Five homogeneous nucleus hives of *A. mellifera iberiensis* (PR-01, PR-05, PR-10, PR-16 and PR-21), whose sister queens were born in 2013, were introduced in May of that same year in one of the experimental apiaries of the CIAPA-IRIAF in Marchamalo (Spain). All of them were managed in the same way (including a treatment with Apitraz, once a year, to comply with the legislation regarding the control of varroosis in Spain).

The sampling scheme started 16 months later (autumn 2014) in order to allow time for the colonies to set and to minimize potential confounding effects caused by the stochastic variation of the parasite populations in the initial stages of the experiment (Fig. 3).

By late November 2015 one of the colonies died (PR-10) and two others displayed no harvesting activity but kept interior bees for a few more weeks (PR-01 and PR-16). The other two colonies were asymptomatic at that time and lasted until the end of the following summer (August 2016).

The samples were selected with the objectives of having a representation of all seasons and, above all, investigating if the latest phases of survival of the colonies displayed different patterns of pathogen diversity than the previous ones. Thus, the selection covered one collection per trimester during, roughly, the first year and two from October 2015 until the collapse of the colonies (Fig. 3).

Twenty five exterior and twenty-five interior honeybees were collected from each colony at the selected dates (Fig. 3). Genomic DNA was separately extracted from the abdomen of each individual using the BS96 DNA Tissue extraction protocol in a BioSprint robot (QIAGEN), as previously described^35^. DNA aliquots from 15 individuals were then combined to obtain pooled DNA samples representing the exterior and interior bees collected at each sampling date. In total there were 80 pooled samples, which means that there was a ≥95% likelihood of detecting a parasite present at prevalence ≥20% over the course of the experiment. DNA concentrations were measured with a spectrophotometer (Nanodrop 2000; Thermo Fisher Scientific) and set at final concentration of approximately 40 ng/ µl for PCR-amplification.

### Primers and PCR amplification

The design of universal primers amplifying the greatest possible number of nosematid, trypanosomatid and neogregarine species was performed with Primer Blast (https://www.ncbi.nlm.nih.gov/tools/primer-blast/) after identifying conserved regions within the alignments of multiple Nosematidae (*SSU* and *Actin*), Leishmaniinae (*SSU* and *RPB1*) and Neogregarinorida (*SSU*) sequences available in GenBank. Expected amplicon sizes were shorter than 300 bp, as required for subsequent Ion-PGM sequencing (Table 3).

**Table 3:** Primers used for PCR amplification (Ion PGM sequencing).

	Gene	Primer name	Sequence	Amplicon length	Annealing T
Nosematids	*SSU*	Nos SSU-F	TGGACTGCTCAGTAATACTCACTT	256	60
		Nos SSU-R	ACTTCCCATAACTGCCTCAGA		
	*Actin*	Nos Actin-F	AAGCYTGTGATGTBGATATYAGA	187	60
		Nos Actin-R	ATWGATCCACCAATCCAKACACT		
Trypanosomatids	*SSU*	Tryp SSU-F2	GGCTACCGTTTCGGCTTTTG	183	66
		Tryp SSU-R2	CTTCATTCCTAGAGGCCGTG		
	*RPB1*	Tryp RPB1-F1	GTGGCTGGAYCTGTGGGAGC	283	66
		Tryp RPB1-R1	GCCRTTGATGAACTTCGCCAC		
Neogregarines	*SSU*	Neog SSU-F	GCGCGCTACACTGATACAC	222	64
		Neog SSU-R	TTGTCCGTATTGTTCACCGGA		

PCRs were performed using the Phusion High-Fidelity DNA Polymerase (Thermo Fisher Scientific), which works at slightly higher annealing temperatures than other polymerases. PCR reactions were performed in 20 μl volumes containing 10.4 μl of H_2_O, 4 μl of 5X Phusion HF Buffer, 0.4 μl of dNTP mix 10 mM, 2 μl of each primer 5 μM, 0.2 μl of Phusion DNA Polymerase and 1 μl of DNA. Cycling conditions were set according to manufacturer’s instructions and consisted of an initial denaturalization at 98 °C for 30 s, followed by 45 cycles of 98 °C for 10 s, 60–66 °C for 30 s (Table 3) and 72 °C for 10 s, and a final extension of 8 min at 72 °C. Negative controls were included in all reactions to monitor for any potential contamination.

To avoid amplification biases in multi-template PCRs, two independent 20 μl reactions were run for each sample. PCRs were checked by 2.0% agarose gel electrophoresis. The resulting amplicons from each sample were pooled, purified with Agentcourt AMPure XP (Beckman Coulter) and quantified with Qubit 2.0 (Thermo Fisher Scientific).

### Ion PGM sequencing

#### Library preparation and sequencing

Barcoded libraries were prepared at the Fundación Pública Galega de Medicina Xenómica (Santiago de Compostela, Spain) by combining equimolar concentrations of the amplicons obtained for each sample with the aid of the Ion Plus Fragment Library Kit and Ion Xpress Barcode Adaptors 1-96 Kit (Thermo Fisher Scientific).

Template preparation and enrichment were carried out on the Ion Chef System (Thermo Fisher Scientific). The template-coated Ion Sphere particles were loaded on an Ion 318 Chip v2 (Thermo Fisher Scientific) and sequenced on an Ion PGM (Thermo Fisher Scientific), which can provide up to 5 million reads/ run, with an average length of 200-300 base pairs^32^.

#### Data analysis

Raw Ion PGM reads were processed with the Ion Torrent Suite software, which scored the quality of the runs and sorted the data according to the barcodes. The assignment of reads to a specific amplicon was made based on the sequences of the primers (which were removed from further analyses). Once identified, these were divided into individual fasta files providing the number and sequence of the haplotypes detected for each amplicon in a sample, as well as the number of reads on each direction (forward and reverse). The latter was used as a control to differentiate sequencing errors from genuine mutations, as indel formation is a frequent and strand-biased artefact of this sequencing technology^62^.

### Evolutionary analyses

Phylogenetic analyses were performed with MEGA7^63^ using both sequences retrieved from GenBank (whole-genome shotgun contigs for trypanosomatids and nucleotides for neogregarines, respectively) and sequences generated during the current study. The best model for each locus was selected by applying the Akaike information criterion (AIC) and the reliability of the resulting tree topologies was tested by bootstrap support (1000 replicates). The evolutionary distances among trypanosomatid sequences (*RPB1*) were estimated using the Tamura 3-parameter model following a gamma distribution whereas those among neogregarine species (*SSU*) were computed using the Kimura 2-parameter method (also following a gamma distribution). Divergence estimates between species were calculated with DnaSP v6^64^.

## Supplementary information

Supplementary Figure S1.

Supplementary Table S1.

Supplementary Table S2.

Supplementary Table S3.

Supplementary Table S4.

Supplementary Table S5.

Supplementary Table S6.

Supplementary Table S7.

## Data Availability

Sequences from previously undescribed parasites were submitted to GenBank (Accession Numbers MN031271.1 and MN038411.1).
