# Supplementary material for: Longitudinal analysis on parasite diversity in honeybee colonies: new taxa, high frequency of mixed infections and seasonal patterns of variation
Source: Sci Rep. 2020 Jun 26;10:10454. doi: 10.1038/s41598-020-67183-3 (PMC7319982; doi:10.1038/s41598-020-67183-3)

Supplementary Figure S1. Relative frequency of positive samples (%) across seasons in interior and exterior bees. Error bars represent 95% confidence intervals.

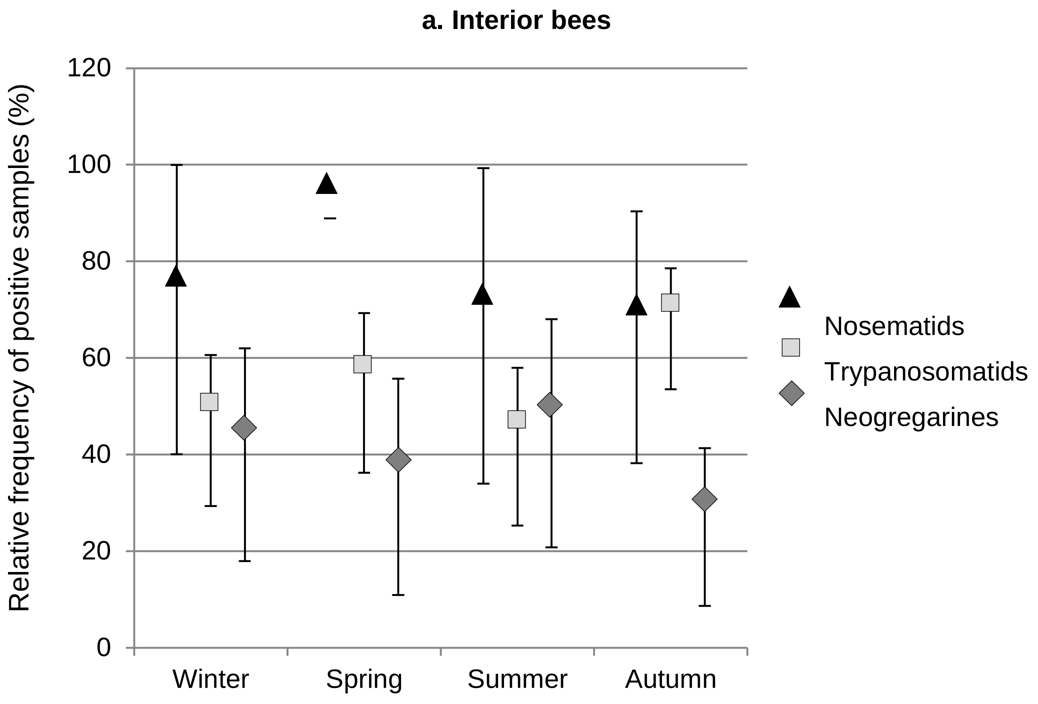

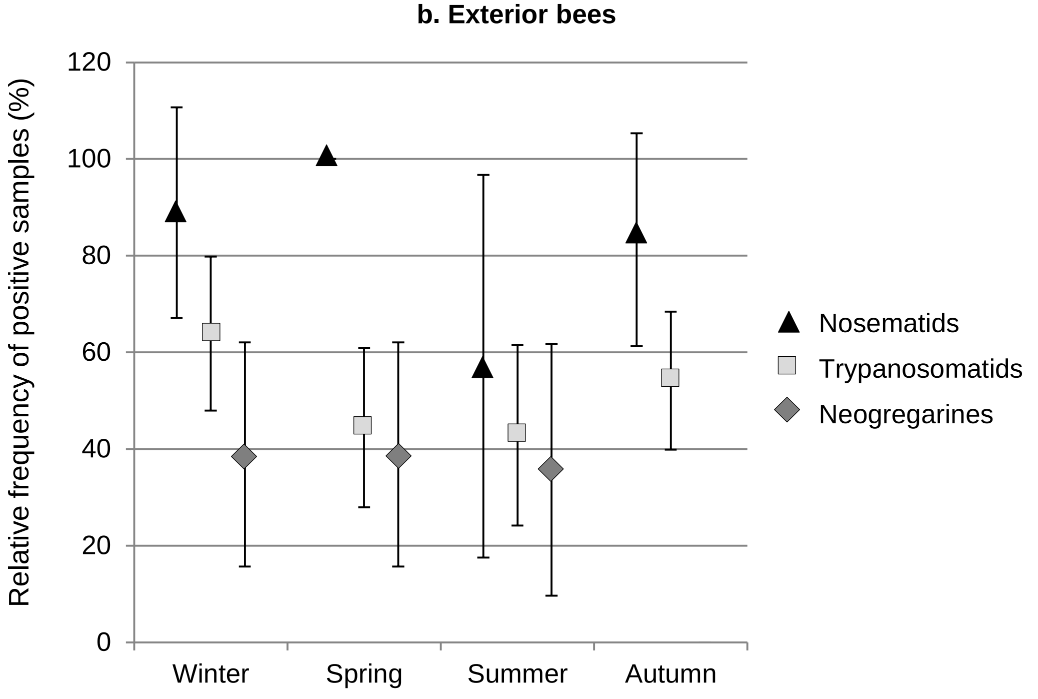

Supplement: Supplementary file 1 — Supplementary Figure S1. [file 41598_2020_67183_MOESM1_ESM.pdf]
